# Employing variance component estimation for point cloud based geometric surface representation by B-splines

**DOI:** 10.1515/jag-2025-0037

**Published:** 2025-05-05

**Authors:** Elisabeth Ötsch, Corinna Harmening, Hans Neuner

**Affiliations:** Research Group Engineering Geodesy, Geodesy and Geoinformation, TU Vienna, Vienna, Austria; Geodetic Sensor Systems, Geodetic Institute, Karlsruhe Institute of Technology, Karlsruhe, Germany

**Keywords:** TLS, B-spline approximation, measurement uncertainty, model uncertainty, variance component estimation

## Abstract

When it comes to the significance assessment of interepochal displacements, great reliance on the stochastic information related to the point clouds introduced in the estimation of the geometric representation of the analysed surface is present. Possible uncertainty sources in point clouds are instrument-, environment- or object sided. Further, the chosen mathematical model for point cloud approximation may introduce an uncertainty budget ascribed as model uncertainty. The present contribution employs variance component estimation (VCE) in the course of geometric point cloud approximation with tensor product B-spline surfaces. A method using the BIQUE-estimation of the variance components is used. It enables considering overlapping variance components. Here, those are related to measurement and model uncertainties. The aim of the article is to investigate whether a realistic estimation of the components can be achieved by means of the VCE in particular with regard to the separation between measurement and model uncertainty. The former include the distance and angular components whilst the model uncertainty is set up using covariance functions. For that, a B-spline surface with comparably superior complexity, whilst describing an identical geometric surface course as the functionally employed surface representation, is generated. Artificial altering of the more complex surface establishes the model uncertainty as distance between equally parametrized sampled points. Results based on simulated data show that variance components are separable and estimable if the model uncertainty exceeds measurement uncertainty, and only points affected by model deviations are included in the VCM setup.

## 1 Introduction

When processing point clouds from terrestrial laser scanning (TLS) to analyse an object’s temporal stability, it is crucial to consider the error budget that comes with TLS data. Soudarissanane et al. [1] divide the error sources as being related to the instrument itself, to the scanning geometry, to the atmosphere the laser beam penetrates and its interaction with the object’s surface. These components lead to systematic as well as random errors. This paper refers to random errors and residual systematics that are denoted as uncertainty conformed by GUM [2]. Uncertainties need to be quantified and further incorporated in the data processing chain by incorporating stochastic information in the form of variance-covariance matrices (VCM). Promising efforts in the context of TLS point clouds have already been undertaken by e.g. [3], [4], [5]. In space-continuous deformation analysis, integrating stochastic information from point clouds is crucial in assessing the significance of interepochal geometric differences. See e.g. M3C2-EP [6], a collocation approach [7], NURBS – resp. B-spline based approaches [8], [9], [10] or also a TLS-enhanced deformation analysis approach [11]. The integration of VCM is necessary in order to bypass type 1 and type 2 errors in a hypothesis test [12].

A preceding step in point cloud analysis is the geometrical modeling of the point clouds. Model selection criteria aid to establish a suitable geometric (functional) model in relation to the underlying data that optimally describe the data generating phenomenon. Herefor, investigations on the determination of a sufficient model’s complexity for tensor product (TP) B-spline surfaces have already been undertaken by [13]. When it comes to the approximation of a point cloud that is impacted by the stated uncertainty sources, the determination of a suited functional model complicates as the exact geometric representation of the actual surface is not known. Systematic deviations arise in the chosen geometric model if it fails to sufficiently capture the geometric details in the point cloud. To address these deviations in the point cloud approximation, the stochastic model can be extended to include an additional source of uncertainty being the model uncertainty.

In this paper TP B-splines are used as functional models for point cloud based surface representations. The approximation by TP B-splines incorporating the stochastic model can be formulated in a Gauss-Markov model (GMM). Therein, the determination of the unknown parameters is performed iteratively given an outlier detection is incorporated. In that course observations are down weighted respectively. As many uncertainty sources are joint in the stochastic model, knowledge of the variance balancing might be absent.

This paper proposes a variance component estimation (VCE) in the course of point cloud based surface approximation using TP B-splines. The presented approach allows the determination of overlapping variance components (VC) with a focus on measurement and model uncertainty. A VCM is established to account for the model uncertainty based on estimated covariance functions. The key novelty of this paper lies in the investigation of the separability of measurement and model uncertainty whilst estimating overlapping VC for their respective uncertainty sources. The proposed procedure is applied on simulated data.

This work is incorporated in the research project TLS-Defo (FOR 5455) aiming at a holistic approach to investigate space-continuous deformation based on point clouds of objects related to the critical infrastructure.

This paper consists of the following sections: In Section 2 the methodology for the consecutive separation of (overlapping) VC in the context of a B-spline surface approximation based on TLS data is introduced. In Section 3 the application of the proposed method on simulated data is explained, followed by the description of the retrieved results in Section 4. After the discussion of the generated results in Section 5, a summary of the proposed study and an outlook on further enhancements is given in Section 6.

## 2 Methodology

### 2.1 Surface representation using TP B-splines

In surface modeling based on point clouds the usage of B-splines allows deriving a statement on (significant) present deformation between consecutively acquired data of an object of interest [7], 9]. A TP B-spline surface is defined as follows:

(1) $$S\left(u,v\right)=\overset{n}{\underset{i=0}{\sum }}\overset{m}{\underset{j=0}{\sum }}N_{i,p}\left(u\right)N_{j,q}\left(v\right)P_{i,j}.$$

It can be regarded as the composition of an infinite number of B-spline curves running in two different parameter directions *u* and *v*. One point **S**(*u*, *v*) on the surface is determined as the weighted sum of the control points **P_i,j_**. The weights are recursively formulated Bernstein polynomials, whose occurrence or elimination depend on the knot vectors **U** resp. **V** and the respective degrees *p* and *q* [14], p. 50]. The net of the control points **P_i,j_** builds the scaffold of the surface. A geometrical change of a point on the surface is linked to a change within the set of connected control points.

### 2.2 Incorporation of the stochastic model in the surface representation

The approximation of a point cloud by a TP B-spline surface can be formulated in a GMM, where the observations **l** are the points' cartesian coordinate elements. The design matrix **A** incorporates the partial derivations of the respective functional model according to the unknowns. An enhancement by the stochastic model in the form of a dispersion matrix **Σ**_**ll**_ is feasible [15]. For the incorporation of the surface approximation formulated in a GMM in a VCE, the observations must not contain any outliers. An approach for robust estimation of B-spline surface parameters is examined in [16]. As this paper is based on simulated data, no outliers are comprised in the data.

### 2.3 VCM comprising measurement uncertainties

The measurement uncertainties impact the noise level of each individual point of a point cloud. The uncertainties concerning the range and angular measurement are typically stated by the manufacturer. The establishment of a VCM incorporating these uncertainties is obtained by error propagation (EP). The functional relation between a point cloud in 3D cartesian space and the measured geometric elements are:

(2) $$\left[\begin{array}{c} x\\ y\\ z \end{array}\right]=\left[\begin{array}{c} r\cdot \sin\left(v\right)\cdot \sin\left(hz\right)\\ r\cdot \sin\left(v\right)\cdot \cos\left(hz\right)\\ r\cdot \cos\left(v\right) \end{array}\right]$$

wherein [*x*,*y*,*z*]^⊺^ are the cartesian coordinate elements of one point, *r* is the measured distance, *hz* the measured horizontal direction and *v* the vertical angle. By applying EP, the VCM **Σ**_*ll*_^*meas*^ of the point cloud’s coordinate elements is set up. In the context of VCE, the VC of the measurement elements *r*, *hz* and *v* shall be estimated. The overlap of the components is outlined in the following with reduction of the equation (2) into 2D space for the sake of easier legibility. The transfer onto equation (2) can be extended straightforward.

(3) $$\left[\begin{array}{c} x\\ y \end{array}\right]=\left[\begin{array}{c} r\cdot \cos\left(hz\right)\\ r\cdot \sin\left(hz\right) \end{array}\right]$$

(4) $$\begin{array}{cc} \Sigma_{ll}^{meas}= & \underset{F}{\underset{︸}{\left[\begin{array}{cc} \cos\left(hz\right) & -r\cdot \sin\left(hz\right)\\ \sin\left(hz\right) & r\cdot \cos\left(hz\right) \end{array}\right]}}\cdot \underset{\Sigma_{ll}}{\underset{︸}{\left[\begin{array}{cc} \sigma_{r}^{2} & 0\\ 0 & \sigma_{hz}^{2} \end{array}\right]}}\cdot \ldots \\ & \underset{F^{⊺}}{\underset{︸}{\left[\begin{array}{cc} \cos\left(hz\right) & \sin\left(hz\right)\\ -r\cdot \sin\left(hz\right) & r\cdot \cos\left(hz\right) \end{array}\right]}}= \end{array}$$

$$\begin{array}{cc} & =\sigma_{r}^{2}\cdot \underset{Q_{ll}^{r}}{\underset{︸}{\left[\begin{array}{cc} \cos{\left(hz\right)}^{2} & \sin\left(hz\right)\cdot \cos\left(hz\right)\\ \sin\left(hz\right)\cdot \cos\left(hz\right) & \sin{\left(hz\right)}^{2} \end{array}\right]}}\\ & \;+\sigma_{hz}^{2}\cdot \underset{Q_{ll}^{hz}}{\underset{︸}{\left[\begin{array}{cc} r^{2}\cdot \sin{\left(hz\right)}^{2} & -r^{2}\cdot \cos\left(hz\right)\cdot \sin\left(hz\right)\\ -r^{2}\cdot \cos\left(hz\right)\cdot \sin\left(hz\right) & r^{2}\cdot \cos{\left(hz\right)}^{2} \end{array}\right]}}\\ & =\sigma_{r}^{2}\cdot Q_{ll}^{r}+\sigma_{hz}^{2}\cdot Q_{ll}^{hz}=\Sigma_{ll}^{r}+\Sigma_{ll}^{hz} \end{array}$$

### 2.4 Deriving empirical VCM from given data

To address the stochastic representation of residual systematics, an empirical VCM of a point cloud relative to a geometric model **S**(*u*, *v*) can be constructed using covariance functions.

The residuals are given as deviations

(5) $$\delta \left(u_{i},v_{i}\right)=\left[\begin{array}{c} \delta_{x}\left(u_{i},v_{i}\right)\\ \delta_{y}\left(u_{i},v_{i}\right)\\ \delta_{z}\left(u_{i},v_{i}\right) \end{array}\right]$$

representing the variability of the point cloud in respect to the model **S**(*u*, *v*). In order to compactly summarize the variability of the points deviations *δ*, empirical autocovariance functions ${\hat{C}}_{xx}\left({\bar{d}}_{k}\right)$ , ${\hat{C}}_{yy}\left({\bar{d}}_{k}\right)$ and ${\hat{C}}_{zz}\left({\bar{d}}_{k}\right)$ as well as crosscovariance functions ${\hat{C}}_{xy}\left({\bar{d}}_{k}\right)$ , ${\hat{C}}_{xz}\left({\bar{d}}_{k}\right)$ and ${\hat{C}}_{yz}\left({\bar{d}}_{k}\right)$ are estimated as exemplarily shown for ${\hat{C}}_{xy}\left({\bar{d}}_{k}\right)$ in equation (6) [17], p. 341ff]:

(6) $${\hat{C}}_{xy}\left({\bar{d}}_{k}\right)=\frac{1}{\left|N_{k}\right|}\sum \delta_{x}\left(u_{i},v_{i}\right)\cdot \delta_{y}\left(u_{j},v_{j}\right)$$

This computation requires a subdivision of the data points’ separation distances *d*_*ij*_ = ‖**S**(*u*_*i*_, *v*_*i*_) − **S**(*u*_*j*_, *v*_*j*_)‖ into consecutive intervals *N*_*k*_ of size |*N*_*k*_|, each with a mean separation distance ${\bar{d}}_{k}$ , as the data points are not equidistant. The chosen approach is similar to the estimation of variogrammes and covariogrammes in the case of collocation [18], p. 131]. In order to ensure a stable estimation, only data pairs with a spatial distance less than or equal half of the diameter of the entire region covered by the incorporated points are taken into account [19], p. 32].

Having estimated the empirical covariance functions, analytical positive semidefinite functions $C_{xx}\left({\bar{d}}_{k}\right),\ldots$ , $C_{yz}\left({\bar{d}}_{k}\right)$ are used to approximate the empirical values and to guarantee positive semidefinite covariance matrices. The covariance matrices are set up by means of the analytical functions as follows:

(7) $$\Sigma_{ll}^{model}=\left[\begin{array}{ccc} \Sigma_{xx} & \Sigma_{xy} & \Sigma_{xz}\\ \Sigma_{yx} & \Sigma_{yy} & \Sigma_{yz}\\ \Sigma_{zx} & \Sigma_{zy} & \Sigma_{zz} \end{array}\right]$$

with the autocovariance Σ_*ii*_ and crosscovariance matrices Σ_*ij*_ being established as follows:

$$\Sigma_{xy}=\left[\begin{array}{cccc} C_{xy}\left(d_{11}\right) & C_{xy}\left(d_{12}\right) & \ldots & C_{xy}\left(d_{1n}\right)\\ C_{xy}\left(d_{21}\right) & C_{xy}\left(d_{22}\right) & \ldots & C_{xy}\left(d_{2n}\right)\\ \vdots & \vdots & \ddots & \vdots \\ C_{xy}\left(d_{n1}\right) & C_{xy}\left(d_{n2}\right) & \ldots & C_{xy}\left(d_{nn}\right) \end{array}\right]$$

### 2.5 Estimation of VC using a best invariant quadratic unbiased estimation (BIQUE)

Alongside the assessment of unknown parameters in a GMM, whose functional model is expressed by (1), the estimation of VC *α*_*i*_ comprised in the VCM **Σ**_*ll*_ of the observations – being the point cloud – is feasible, provided that a parametrized stochastic model is implemented [15], p. 227ff]:

(8) $$\Sigma_{ll}=\overset{m}{\underset{i=1}{\sum }}\alpha_{i}^{2}\cdot \Sigma_{i}.$$

The parameter $\alpha_{i}^{2}$ is the factor of the variance resp. covariance to be estimated. The parameter *m* is the number of (co-)variance components that are assessed and accordingly, the number of splitted covariance matrices that form the overall VCM of the observations **l** [15], p. 227ff].

The estimation of VC is a generalization of the assessment of the variance of the unit weight $s_{0}^{2}=\frac{v^{⊺}Pv}{n_{f}}$ where $P=\Sigma_{ll}^{-1}$ . Likewise to the estimation of the unknowns in the GMM, the determination of the VC follows a best unbiased estimation given that

(9) $$E\left\{l^{⊺}Dl\right\}=p^{⊺}\cdot \alpha .$$

The expectancy value *E* of the quadratic sum of the observations weighted by the estimator matrix **D** is set equal to the scalar form **p**^⊺^ ⋅ *α*, whereas $\alpha ={\left[\alpha_{1}^{1},\alpha_{2}^{2},\ldots ,\alpha_{m}^{2}\right]}^{⊺}$ is the vector of estimated VC and **p** is used as vector with logical entries indicating the estimation of one specific variance component $\alpha_{i}^{2}$ . Given the added condition that the estimation minimizes the variance

(10) $$\mathrm{Var}\left\{l^{⊺}Dl\right\}=\mathrm{Var}\left\{p^{⊺}\cdot \alpha \right\}\to \min,$$

[20] introduces VCE using a BIQUE approach. For this, an additional constraint must be satisfied, requiring the invariance of the VC *α* w.r.t. the unknown parameters. This constraint is expressed as **D** ⋅ **A** = **0** and *p*_*i*_ = tr(**D** ⋅ **Σ**_*i*_), where **A** is the design matrix setup in the GMM.

By derivation of equation (9) and imposing the additional condition (10) and the constraint on independence from the parameter space, one obtains according to [20]

(11) $$W=\Sigma_{ll}^{-1}\Sigma_{vv}\Sigma_{ll}^{-1}$$

(12) $$q_{i}=v^{⊺}\left(P\left(\Sigma_{i}\cdot \alpha_{i}^{2}\right)P\right)v$$

(13) $$s_{i,j}=\text{tr}\left(W\left(\Sigma_{i}\cdot \alpha_{i}^{2}\right)W\left(\Sigma_{j}\cdot \alpha_{j}^{2}\right)\right).$$

The estimation of the VC is retrieved by

(14) $$\hat{\alpha }=S^{-1}\cdot q.$$

The VC are estimated iteratively. The starting values for all $\alpha_{i}^{2}$ are set to 1. The determination of the VC $\hat{\alpha }$ is local which means it is a prerequisite that the factors $\alpha_{i}^{2}$ are near 1 in order to achieve convergence in an iterative estimation process [15], p. 227ff].

## 3 Simulation studies

In the presented simulation studies a VCE in the course of a point cloud based TP B-spline approximation is investigated.

### 3.1 Conceptional considerations

**Figure 1:**
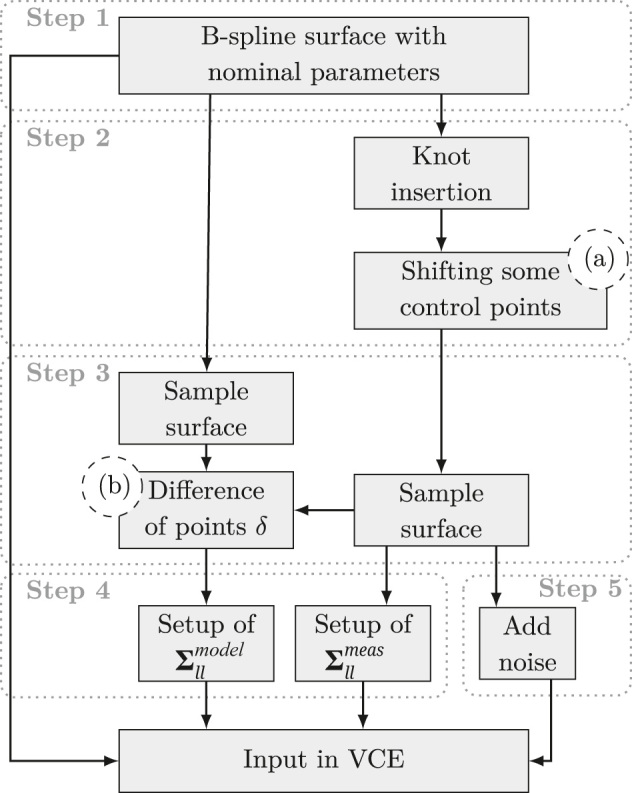
Workflow for the setup of the simulation study. Indicators (a) and (b) refer to Figure 2. The respective steps are discussed in detail in Sections 3.1 and 3.2.

As a first step towards the application of the VCE in the context of B-spline surface approximation, simulation studies are conducted. In Figure 1 the steps of the setup of the study are displayed.

A TP B-spline surface of well-known shape and parameters is used as geometrical (functional) model (Figure 1, Step 1). In the following this surface will be referred to as initial surface **S**_1_(*u*, *v*). On basis of this surface, the simulation of the model uncertainty is conducted.

The model uncertainty is considered as the deviation that occurs when the chosen complexity of the functional model, used in the surface approximation, does not adequately reflect the geometric details contained in the point cloud. For TP B-splines, complexity refers to the number of control points.

The deviation of a point cloud to the model **S**_1_(*u*, *v*) is realised by increasing it’s complexity without changing it’s course. This is done by knot resp. control point insertion in selected locations [14], p. 141ff] (Figure 1, Step 2). In a next step, a part of the inserted control points $P_{i,j}^{\text{sel}}$ are shifted in the direction of the line-of-sight $\hat{r}$ from a specified instrument center **X**_*ST*_. The amount of displacement of the altered control points $P_{i,j}^{\text{altered}}$ is scaled by a respectively randomized value $\epsilon_{i,j}^{\text{sel}}∼$ N (0,1) multiplied by the magnitude factor *m*_*f*_.

(15) $$P_{i,j}^{\text{altered}}=P_{i,j}^{\text{sel}}+\hat{r}\cdot \left(\epsilon_{i,j}^{\text{sel}}\cdot m_{f}\right)\;\;\text{with }\hat{r}=\frac{P_{i,j}^{\text{sel}}-X_{ST}}{\left\|P_{i,j}^{\text{sel}},-,X_{ST}\right\|}$$

The resulting manipulated surface is referred to as adapted surface **S**_2_(*u*, *v*). By sampling both surfaces with identical parameter spacing and location, two point clouds are generated that provide a point-to-point relation $\left(S_{1}\left(u_{i},v_{i}\right)\overset{\wedge }{=}S_{2}\left(u_{i},v_{i}\right)\right)$ (Figure 1, Step 3). The derived point pairs’ differences are treated as the residuals to the initial surface as *δ* = **S**_1_(*u*, *v*) − **S**_2_(*u*, *v*). Following the setup stated in Section 2.4, the VCM **Σ**_*ll*_^*model*^ is established covering the model uncertainty (Figure 1, Step 4). As the estimation’s stability of the covariance functions depends on the spatial distance of the used points put in one interval *N*_*k*_, the spatial extent of the introduced surface deviations is locally restricted w.r.t. the course of the point cloud. Moreover, for the determination of the model uncertainty, only the point correspondences with *δ*_*i*_ > *θ*, exceeding the other (subsequently) integrated error sources indicated by a threshold *θ*, are taken into account. For the generation of the VCM covering the measurement uncertainty, the sampled points of the adapted surface **S**_2_(*u*, *v*) are transformed in spherical coordinates with respect to the simulated instrument center **X**_*ST*_. Chosen variances of the spherical coordinate components are re-transformed to cartesian coordinates by EP, obtaining the VCM **Σ**^*meas*^_*ll*_ (Figure 1, Step 4). The sampled points were perturbed by adding noise with predefined variances different to those chosen in the EP (Figure 1, Step 5). If the variances of the applied noise are equal to those introduced in the EP, the estimation of the VC would resolve immediately. The difference of the chosen variances enable the investigation of the convergence behaviour of the iterative estimation.

The subsequent VCE is performed by incorporating the sampled and perturbed point cloud of **S**_2_(*u*, *v*), the joint VCM **Σ**_*ll*_ = **Σ**_*ll*_^*meas*^ + **Σ**_*ll*_^*model*^ and the functional model of the initial surface **S**_1_(*u*, *v*).

### 3.2 Numerical setup

The simulation study is realised on the basis of a TP B-spline surface representing the course of a water dam being the test specimen of the research conducted within the project TLS-Defo. The initial surface **S**_1_(*u*, *v*) is parametrized by 30 × 10 (*u* × *v*) control points and is of degree *p*, *q* = 3 (Figure 1, Step 1). The adapted surface **S**_2_(*u*, *v*) is generated by knot resp. control point insertion (Figure 1. Step 2). The sampled point clouds comprise 3,000 single points evenly spread over the course of the surfaces (Figure 1, Step 3). For the setup of **Σ**_*ll*_, the noise levels of the measurement elements are chosen as *σ*_*r*_ = 1 mm and *σ*_*hz*,*v*_ = 0.3 mgon (Figure 1, Step 4). The standard deviations introduced in the EP for the setup of **Σ**^*meas*^_*ll*_ differ from the applied standard deviations with *σ*_*r*_ = 2 mm and *σ*_*hz*,*v*_ = 1 mgon (Figure 1, Step 5).

**Figure 2:**
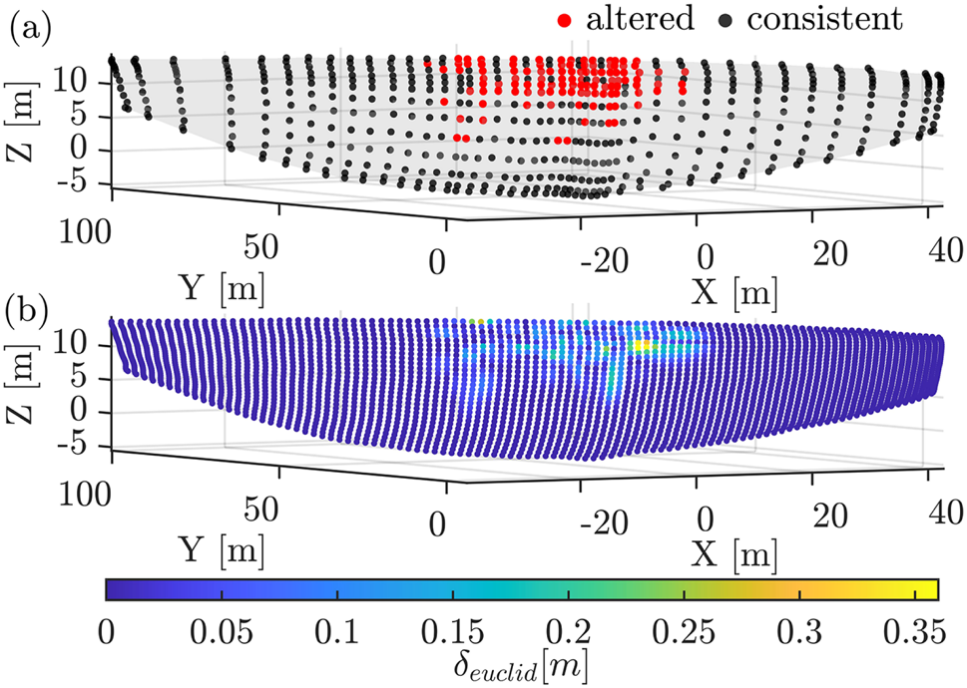
Visualisation of indicators in Figure 1: (a) B-spline surface with control points; (b) sampled points colored by differences *δ*.

For the specification of model uncertainty, the location of the inserted and altered control points as well as the magnitude factor *m*_*f*_ of the displacement is varied. An example of one realisation is shown in Figure 2.

## 4 Results

### 4.1 Monte-Carlo simulation of the measurement uncertainties

To generate a representative result of the simulation study, a Monte-Carlo Simulation (MCS) with multiple realisations of the measurement noise was conducted. In total 600 runs of the VCE were assessed, whereas in each run *i* an independent setup of the measurement noise was realised by

(16) $${\left[\begin{array}{c} r^{\prime }\\ hz^{\prime }\\ v^{\prime } \end{array}\right]}_{i}={\left[\begin{array}{c} r\\ hz\\ v \end{array}\right]}_{i}+{\left[\begin{array}{ccc} \epsilon_{r} & 0 & 0\\ 0 & \epsilon_{hz} & 0\\ 0 & 0 & \epsilon_{v} \end{array}\right]}_{i}\cdot \left[\begin{array}{c} \sigma_{r}\\ \sigma_{hz}\\ \sigma_{v} \end{array}\right]$$

where *ϵ*_*r*_, *ϵ*_*hz*_, *ϵ*_*v*_ ∼ N (0,1) are independent random variables drawn from a standard normal distribution. The VCM of the model uncertainty **Σ**_*ll*_^*model*^ was fixed for all runs and was built upon the differences of the sampled surface points of the initial and adapted surface. However, solely points with coordinate-wise differences |*δ*_*x*_| > *θ* or |*δ*_*y*_| > *θ* with *θ* = 4 mm are used. The specification of the threshold value *θ* was justified by being twice the magnitude of the mean standard deviation of the lateral components of the perturbed point cloud. Based on the totality of all results of the computed VCE, the mean value and the standard deviation of the estimated variance factors *α*_*i*_ could be determined and are stated in Table 1. For better interpretability, the VC are multiplied with the initially applied variances inserted in the VCM and transformed into standard deviations. They are opposed to the reference values, being the factors of standard deviations that were initially applied to the data.

**Table 1:** Variation of estimated VC in a Monte Carlo simulation.

$\sqrt{\alpha_{i}\cdot \sigma_{i}^{2}}$	Reference	mean	std
Distance [mm]	1	1.1	0.02
Horizontal direction [mgon]	0.3	0.3	0.005
Vertical angle [mgon]	0.3	0.3	0.005
Model uncertainty []	1	0.9	0.003

### 4.2 Influence of the magnitude of surface deviations on the estimated VC

The setup of the VCM **Σ**_*ll*_^*model*^ covering the model uncertainty is influenced by two driving factors. Firstly, the location of the displaced control points forming separated regions of deviations. Secondly, the magnitude of the displacements expressed as the magnitude factor in equation (15). This section focuses on the second aspect whilst the first is addressed in the Annex.

**Figure 3:**
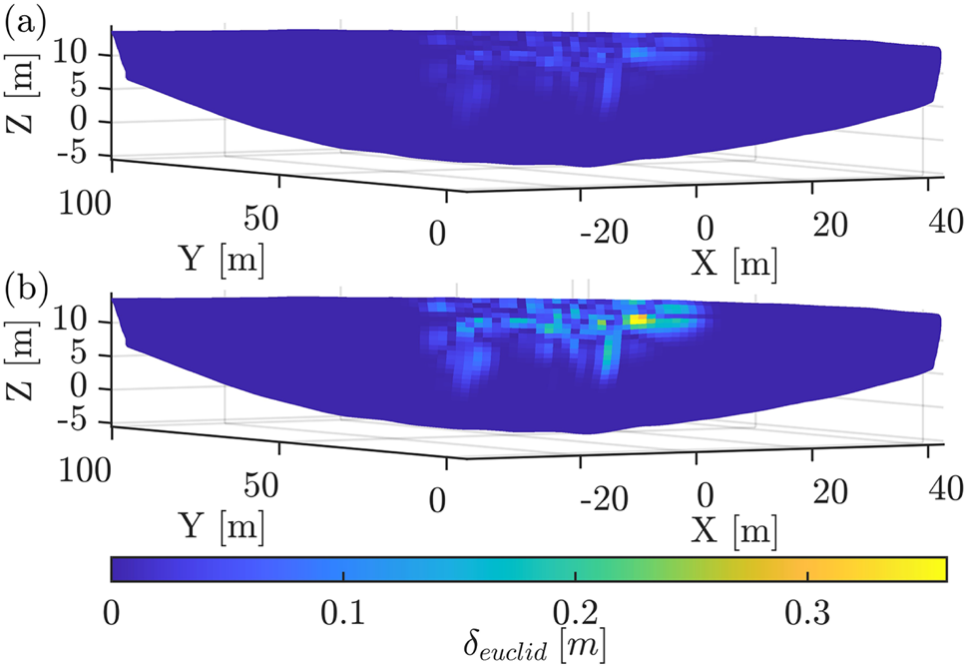
Surfaces colored by *δ* based on (15) with (a) *m*_*f*_ = 0.06 m, (b) *m*_*f*_ = 0.2 m.

**Figure 4:**
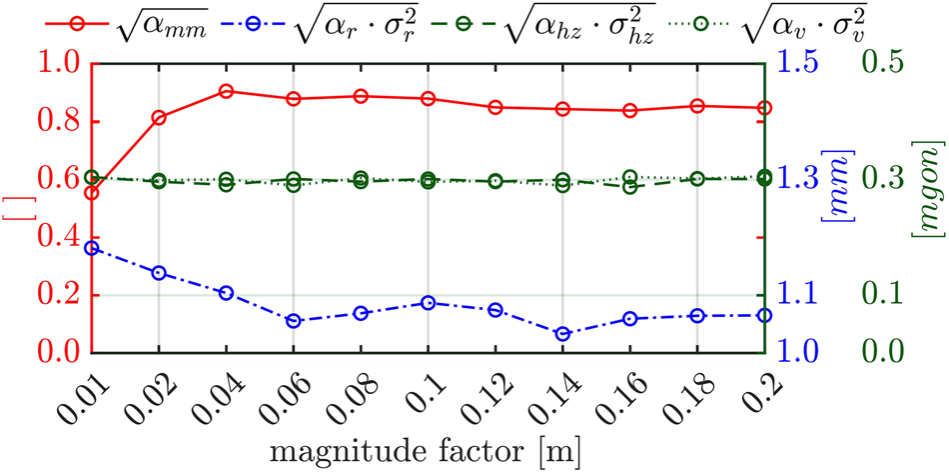
Course of the estimated VC related to a variation of *m*_*f*_ of a fixed selection of control points and a consistent pattern of displacement.

In Figure 3 the displacement between ***S***_1_(*u*, *v*) and ***S***_2_(*u*, *v*) is shown for two realisations. The pattern of the displacement is consistent. For both cases the selection of the altered control points and the respectively randomized value *ϵ* are fixed. Solely the magnitude factor *m*_*f*_ is different. To investigate the impact of the magnitude factor *m*_*f*_ on the estimated VC, 11 realisations of the surface deviations are created in reference to the deviation pattern in Figure 3. Herein, *m*_*f*_ ranged from 0.01 to 0.2 m. If the magnitude of displacement is in the range of the propagated measurement uncertainty, affecting the whole point cloud, the separation between model and measurement uncertainty aggravates. This behaviour is displayed in Figure 4.

On the abscissa, the magnitude factor for the displacement of a set of control points is plotted. On the ordinate the derived standard deviations impacted by the estimated VC are displayed. The displacement of the control points are performed in the line-of-sight of the simulated scanner position. Hence, it impacts mainly the variance component related to the distance component (blue/dot-dashed) and the model uncertainty (red/solid). The VC related to the angular measurement elements are consistent throughout all estimations (green/dashed resp. dotted). The integrated model uncertainty is intended to be fully depicted in the VCM **Σ**_*ll*_^*model*^. Hence, the expected variance component is 1. However, the estimated variance component changes with the variation of *m*_*f*_. The course of the value change reaches saturation at *σ*_*mm*_ = 0.8 − 0.85.

## 5 Discussion

The presented results of the realised simulation studies state an excerpt of possible realisations. Restrictions as well as possible enhancements of the proposed method and the implemented simulation studies shall be discussed in the following:

Regarding the setup of **Σ**_*ll*_^*model*^, the introduced deviations of the point cloud relative to the model are reasonable as only points affected by the artificial displacement of control points are considered in the estimation of the covariance functions. Including all points in the estimation would strongly bias the resulting stochastic model due to the influence of points unaffected by the model deviations. This manifests in the fact that model uncertainty is falsely introduced for those points. In the VCE procedure, this causes the respective variance component to become negative when holding the assumption that the starting value for *α*_*i*_ is near 1. The restricted incorporation of exclusively points that are affected by the introduced model uncertainty dissolves this problem. In the present case a threshold *θ* is set to divide the point cloud accordingly. The chosen threshold is related to the noise level of the point cloud accounting for the measurement uncertainties. This can be transferred to the application on other cases, as the setup of the VCM of the measurement uncertainty is straight forward by using the instrument’s specification.

The presented results show that the estimation of the covariance functions is strongly dependent on the location and the magnitude of the incorporated points. Here, the model deviations are restricted to a local region on the surface. If this is not the case and the patches of point deviations are spread throughout the course of the whole surface, the estimation of the respective covariance functions gets unstable leading to separation problems of VC.

Moreover, **Σ**_*ll*_^*model*^ is simplified here as solely autocovariances between the respective coordinate elements are introduced. Currently, crosscovariances are neglected, as their inclusion was found to increase the deviation of the estimated variance component from the expected value of 1. This aspect is here declared as open and shall be investigated in future research work.

Additionally, it should be noted that only two uncertainty sources are considered in the VCE. Certainly, the uncertainty sources that contribute to acquired point clouds are manifold. The expansion of the proposed methodology by other uncertainty sources is considered straightforward and shall be realised in subsequent work.

## 6 Conclusions

This paper introduces a VCE for the TP B-spline approximation of point clouds. The set focus for the current case studies lied in the separability of measurement and model uncertainty whilst employing the VC for the respective uncertainty sources. The enhancement to other uncertainty sources in the procedure remains open for follow-on research work. Simulation studies based on a TP B-spline surface showing the course of a water dam were realised. The application of the measurement uncertainty was conducted by adding noise. The model uncertainty was introduced starting from an initial surface, increasing its complexity by adding control points and finally modifying their location. The functional model corresponding to the initial B-spline surface is employed for the surface approximation. The setup of the respective stochastic models were conducted on the one hand by using EP and on the other by the estimation of covariance functions. The further assessment of the VC was realised in a BIQUE approach. The results show, that under certain, priorly stated conditions, the VC are estimable within the simulation studies. The application of the approach on real acquired data remains open as a future research scope.

## Figures and Tables

**Figure 5:**
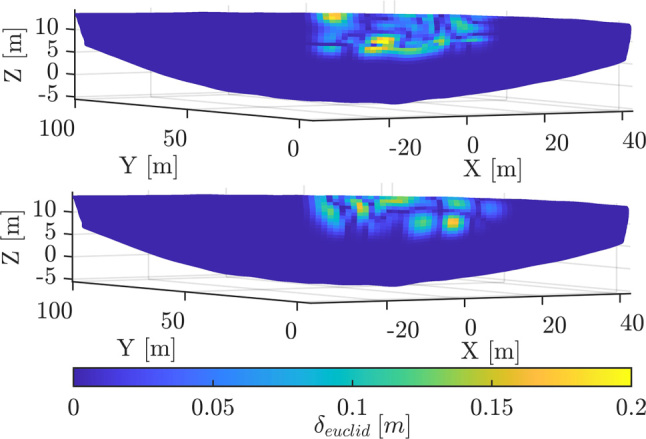
Surface colored by euclidean differences between corresponding surface points establishing the model uncertainty in varying restricted location with consistent magnitude.

## Annex: Additional results

### Investigation of the impact of the location of the altered control points to generate the model uncertainty

In contrast to the previous process, the magnitude factor *m*_*f*_ = 0.2 remains constant here. Solely the randomized value *ϵ*, drawn from the standard normal distribution N (0,1), is regenerated consecutively. In Figure 5 two examples for realisations are displayed.

In total 50 runs were performed. The mean value and it’s standard deviation of all estimated VC are shown in Table 2. In contrast to the estimated variance component of the measurement elements, the variance component related to the model uncertainty varies distinctively with the change of the applied displacement pattern generated by *ϵ*.

**Table 2:** Variation in estimated VC based on changes in the displacement pattern for model uncertainty.

$\sqrt{\alpha_{i}\cdot \sigma_{i}^{2}}$	Reference	mean	std
Distance [mm]	1	1.0	0.02
Horizontal direction [mgon]	0.3	0.30	0.004
Vertical angle [mgon]	0.3	0.30	0.005
Model uncertainty []	1	0.66	0.15
